# Early Aortic Valve Replacement in Moderate Aortic Stenosis

**DOI:** 10.1016/j.jacadv.2024.101190

**Published:** 2024-08-16

**Authors:** Omar M. Abdelfattah, Xander Jacquemyn, Samir R. Kapadia, Nicholas Van Meighem, Marie-Annick Clavel, Philippe Généreux, Martin Leon, Philippe Pibarot

**Affiliations:** a University of Texas Medical Branch, Galveston, Texas, USA; b KU Leuven, Leuven, Belgium; c Heart, Thoracic and Vascular Institute, Cleveland Clinic, Cleveland, Ohio, USA; d Erasmus University Medical Center, Rotterdam, the Netherlands; e Quebec Heart & Lung Institute, Laval University, Quebec City, Quebec, Canada; f Gagnon Cardiovascular Institute, Morristown Medical Center, Morristown, New Jersey, USA; g Clinical Trials Center, Cardiovascular Research Foundation, New York, New York, USA; h NewYork-Presbyterian Hospital/Columbia University, Medical Center, New York, New York, USA

**Figure:**
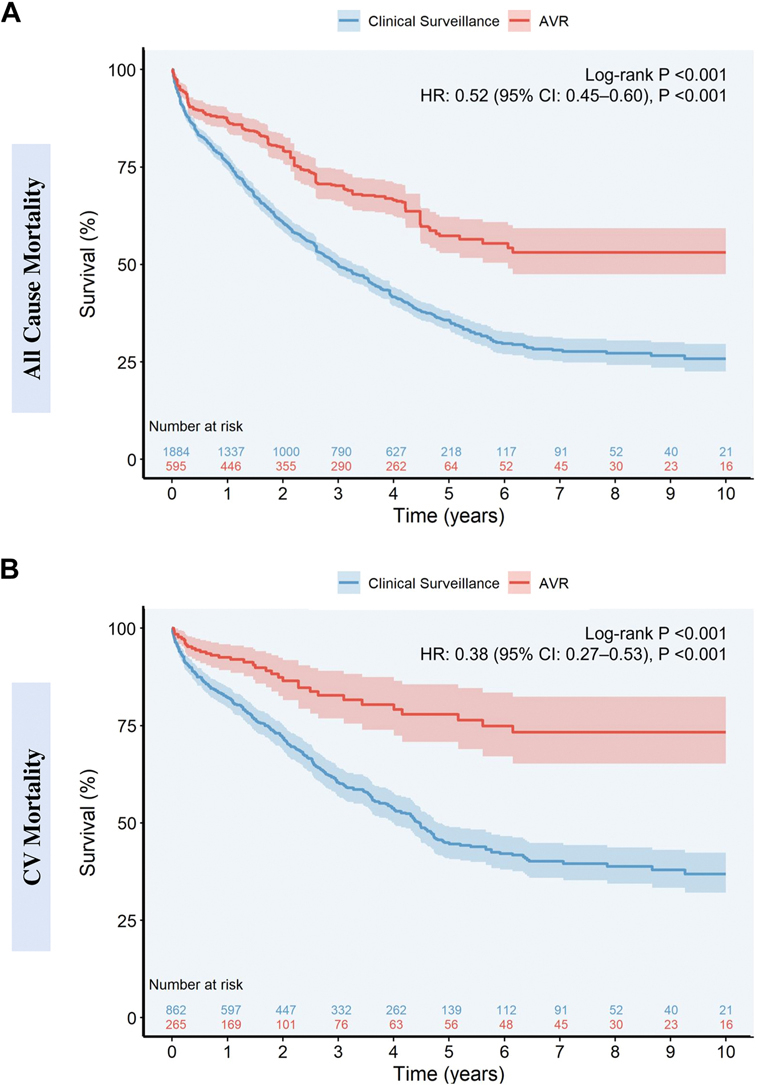

Aortic valve replacement (AVR) is only indicated in patients with moderate aortic stenosis (AS) when another indication coexists for open heart surgery (ie coronary artery bypass, other valve disease, aortopathy). Recent data consistently showed that moderate AS is associated with a high risk of mortality, especially in patients with heart failure and reduced ejection fraction.^1^^,^^2^ Such observations led to the design of the TAVR UNLOAD (Transcatheter Aortic Valve Replacement to Unload the Left Ventricle in Patients With Advanced Heart Failure; NCT02661451) investigating the benefit of AVR in moderate AS with reduced left ventricular ejection fraction (LVEF). Hence, we sought to perform a reconstructed Kaplan-Meier meta-analysis to compare AVR to clinical surveillance in moderate AS with LVEF ≤50%.

This meta-analysis was performed according to the Preferred Reporting Items for Systematic Reviews and Meta-Analysis (PRISMA) guidelines and was prospectively registered in the PROSPERO database (CRD42023483420). Electronic data sets were systematically searched from inception to November 10, 2023, by 2 independent investigators (O.M.A. and X.J.) using the key terms “aortic valve stenosis,” and “moderate,” with no language restrictions. Disagreements were resolved by consensus with a third investigator (P.P.). Inclusion criteria included: 1) observational studies of patients with moderate AS; 2) reduced ejection fraction at baseline (LVEF ≤50%); 3) populations were divided into 2 treatment groups (AVR versus clinical surveillance); and 4) time to event follow-up data were present for at least >2 years. The primary outcome of interest was all-cause mortality. The secondary outcome was cardiovascular mortality. Individual patient data based on published Kaplan-Meier graphs from included studies were reconstructed using the “curve approach.” Two investigators assessed the reconstructed patient data accuracy at each read-in point. In the Kaplan-Meier–based meta-analysis, the mean ± SD survival times, median (IQR) survival times, and survival percentage at different time points with 95% CIs were calculated. The differences in survival between the groups were assessed using the log-rank test for differences and a Cox proportional hazards regression model. Truncated survival analysis at longest follow-up was performed as a prespecified outcome and respective HRs and 95% CI were calculated. Sensitivity analysis was performed for adjusted cohorts. *P* values were 2-sided, and statistical significance was set at *P* < 0.05. All analyses were completed with R, version 4.2.1 (Foundation for Statistical Computing).

**Figure 1:**
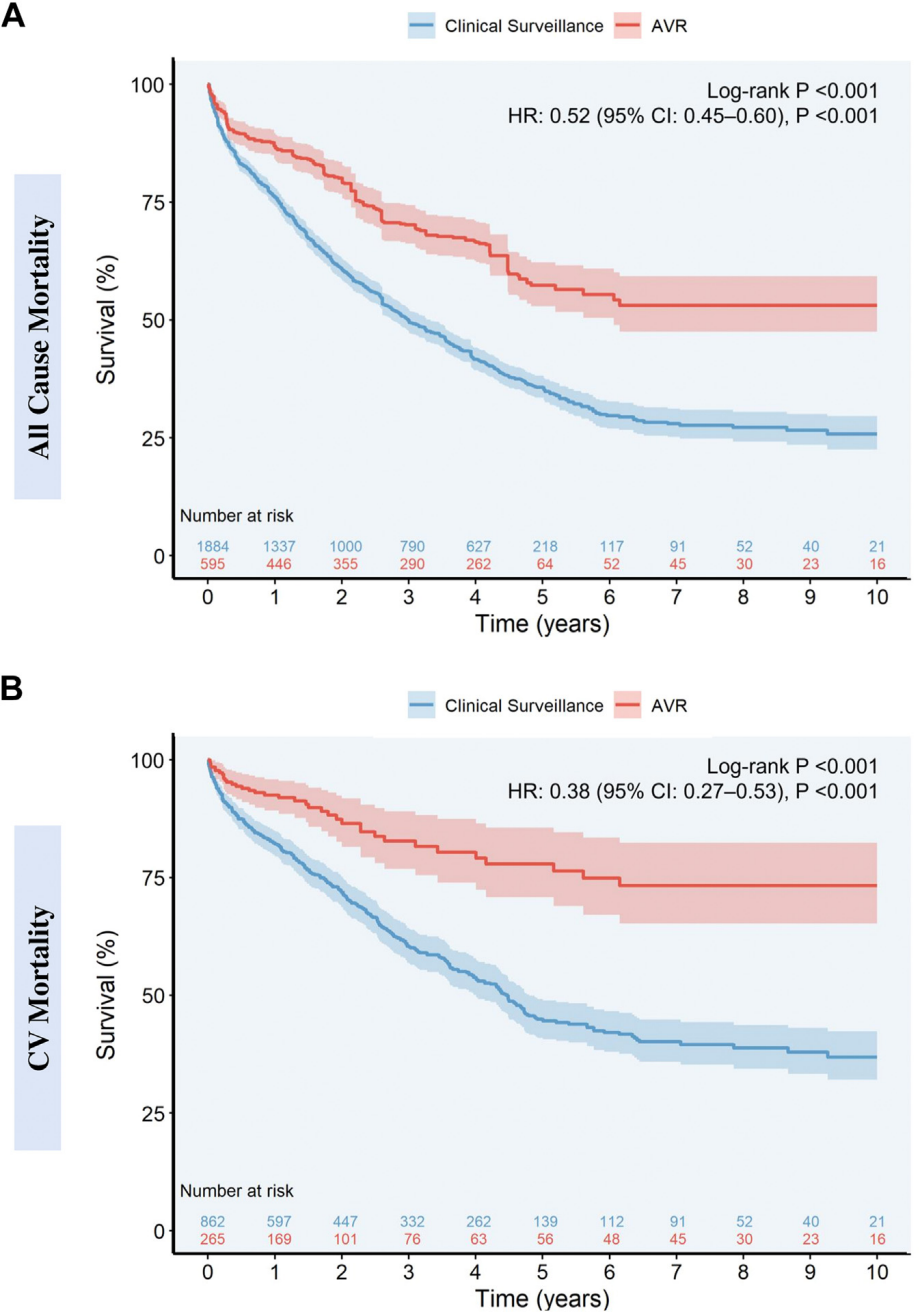
Mortality According to Aortic Valve Replacement Versus Clinical Surveillance in Patients With Moderate Aortic Stenosis and Reduced Ejection Fraction Reconstructed Kaplan-Meier analysis in patients with moderate aortic stenosis and reduced left ventricular dysfunction undergoing aortic valve replacement versus clinical surveillance: (A) All-Cause Mortality, (B) Cardiovascular Mortality. AS = aortic stenosis; AVR = aortic valve replacement; HFrEF = heart failure with reduced ejection fraction.

Five observational studies (n = 2,479, AVR; n = 595 [24%] and clinical surveillance; n = 1,884 [76%]) with 2.4 years (IQR: 0.8–5.0 years) median follow-up were included.^1^, ^2^, ^3^, ^4^, ^5^ The mean age was 75.7 ± 9.8 years in the AVR cohort and 74.3 ± 11.4 years in the clinical surveillance cohort. Male patients constituted 63.3% and 67.9% in the AVR and clinical surveillance groups, respectively. Ten-year overall survival rates among patients with moderate AS and reduced LVEF undergoing AVR compared to clinical surveillance were 53.1% (95% CI: 47.5%–59.3%) and 25.8% (95% CI: 22.6%–29.6%), respectively. AVR was associated with a decreased risk of all-cause mortality (HR: 0.52; 95% CI: 0.44-0.60; *P* < 0.001) (proportional hazards, Schoenfeld residual *P* = 0.55) (Figure 1A). On sensitivity analysis of adjusted studies (n = 3)^1^^,^^4^^,^^5^, 825 patients (AVR; n = 234 and clinical surveillance; n = 591) with 1.6 years (IQR: 0.6–2.6 years) follow-up were included. Seven-year overall survival rates among those with moderate AS undergoing AVR compared to clinical surveillance were 54.6% (95% CI: 44.4%–67.1%) and 22.6% (95% CI: 17.4%–29.3%), respectively. AVR was associated with a decreased risk of all-cause mortality (HR: 0.45; 95% CI: 0.33-0.61; *P* < 0.001) (proportional hazards, Schoenfeld residual *P* = 0.98). Moreover, to account for immortal time bias, we performed a sensitivity analysis following the exclusion of studies with no clear definition of enrollment time for both groups. AVR remained associated with a decreased risk of all-cause mortality (HR: 0.47; 95% CI: 0.34-0.67; *P* < 0.001).

For cardiovascular mortality, a total of 3 observational studies (n = 1,127, [AVR; n = 265 and clinical surveillance; n = 862]) with 1.9 years (IQR: 0.7–4.5 years) follow-up were included in the analysis.^3^, ^4^, ^5^ Ten-year cardiovascular survival rates among those with moderate AS undergoing AVR compared to clinical surveillance were 73.3% (95% CI: 65.2%–82.4%) and 36.9% (95% CI: 32.1%–42.4%), respectively. AVR was associated with a decreased risk of cardiovascular mortality (HR: 0.38; 95% CI: 0.27-0.53; *P* < 0.001) (proportional hazards, Schoenfeld residual *P* = 0.80) (Figure 1B). On sensitivity analysis of adjusted studies (n = 2), 563 patients (AVR; n = 191 and clinical surveillance; n = 372) with 1.4 years (IQR: 0.5–2.0 years) follow-up were included. Seven-year overall survival rates among those with moderate AS undergoing AVR compared to clinical surveillance were 72.3% (95% CI: 60.0%–87.1%) and 44.9% (95% CI: 36.5%–55.3%), respectively. AVR was associated with a decreased risk of cardiovascular mortality (HR: 0.37; 95% CI: 0.24-0.58; *P* < 0.001) (proportional hazards, Schoenfeld residual *P* = 0.64).

This Kaplan Meier (KM)-reconstructed meta-analysis of 5 observational studies with 2,479 moderate AS patients with LVEF ≤50% demonstrated that AVR (transcatheter or surgical AVR) is associated with a significantly lower risk of all-cause and cardiovascular mortality, compared to clinical surveillance. However, given the observational nature of the included studies, caution in the interpretation of results is warranted given the possible unaddressed confounders that might introduce bias. Further long-term and randomized data are required to confirm the benefits of early AVR in this unique population and its impact on long-term outcomes. Several limitations are worth mentioning, including the susceptibility to selection bias due to procedural patient selection and referral bias in high-volume centers. Immortal time bias was accounted for in the sensitivity analysis where time zero for AVR was within 90 days from index echocardiography with moderate AS diagnosis.
